# Understanding the molecular basis of plant growth promotional effect of *Pseudomonas fluorescens *on rice through protein profiling

**DOI:** 10.1186/1477-5956-7-47

**Published:** 2009-12-24

**Authors:** Saveetha Kandasamy, Karthiba Loganathan, Raveendran Muthuraj, Saravanakumar Duraisamy, Suresh Seetharaman, Raguchander Thiruvengadam, Balasubramanian Ponnusamy, Samiyappan Ramasamy

**Affiliations:** 1 Centre for Plant Protection Studies, Tamil Nadu Agricultural University, Coimbatore, India; 2 Centre for Plant Molecular Biology, Tamil Nadu Agricultural University, Coimbatore, India

## Abstract

**Background:**

Plant Growth Promoting Rhizobacteria (PGPR), *Pseudomonas fluorescens *strain KH-1 was found to exhibit plant growth promotional activity in rice under both *in-vitro *and *in-vivo *conditions. But the mechanism underlying such promotional activity of *P. fluorescens *is not yet understood clearly. In this study, efforts were made to elucidate the molecular responses of rice plants to *P. fluorescens *treatment through protein profiling. Two-dimensional polyacrylamide gel electrophoresis strategy was adopted to identify the PGPR responsive proteins and the differentially expressed proteins were analyzed by mass spectrometry.

**Results:**

Priming of *P. fluorescens*, 23 different proteins found to be differentially expressed in rice leaf sheaths and MS analysis revealed the differential expression of some important proteins namely putative p23 co-chaperone, Thioredoxin h- rice, Ribulose-bisphosphate carboxylase large chain precursor, Nucleotide diPhosphate kinase, Proteosome sub unit protein and putative glutathione S-transferase protein.

**Conclusion:**

Functional analyses of the differential proteins were reported to be directly or indirectly involved in growth promotion in plants. Thus, this study confirms the primary role of PGPR strain KH-1 in rice plant growth promotion.

## Background

PGPR has promotional effect on plant growth and developmental processes in two different ways viz., 1) indirectly by decreasing or preventing some of the deleterious effects of a phytopathogenic organism; 2) directly by promoting plant growth through facilitating the uptake of nutrients from the environment [1]. Effect of PGPR on plant growth processes include, increase in germination rates, root growth, leaf area, chlorophyll content, magnesium, nitrogen and protein content, hydraulic activity, tolerance to drought and salt stress, shoot and root weights and delayed leaf senescence [2]. PGPR mediated plant growth enhancement was reported by many workers [3, 4, 1, 5, 6, 7 & 8]. Our previous reports also revealed the growth promotional activity of *P. fluorescens *in rice under laboratory, glass house and field conditions. However, there is no information available on the molecular basis of host plant - PGPR interaction in promoting plant growth.

Among the various molecular biological techniques available, high throughput whole genome gene expression tools viz., microarrays and proteomics will allow us to have improved knowledge on the gene(s) and pathways induced during host-PGPR interaction. 2D-PAGE strategy has been widely used in understanding stress responses as well as in understanding constitutive differences between developmental stages or genotypes. First it provides the broad overview of proteins produced by both the partners. Second it allows the detection of signal transduction pathways and post-translational modifications of proteins, which decides the function of the protein. Recently, Shoresh and Harman [9] characterized *Trichoderma harzianum *and maize interactive proteins and reported the metabolic pathways induced by *T. harizianum*.

The present proteomic study was being carried out to dissect the molecular events induced or affected during rice-*Pseudomonas *interactions. Efficacy of *P. fluorescens *strain KH-1 in promoting plant growth in rice under glass house and field conditions was studied. The study demonstrated the promotional activity of *P. fluorescens *strain KH-1 on rice plant growth and yield [10]. 2D-PAGE analysis of leaf sheaths collected from control and PGPR treated plants revealed the induction of few key proteins involved in key energy metabolism.

## Results

### Effect of *P. fluorescens *on growth parameters in rice

**Figure 1:**
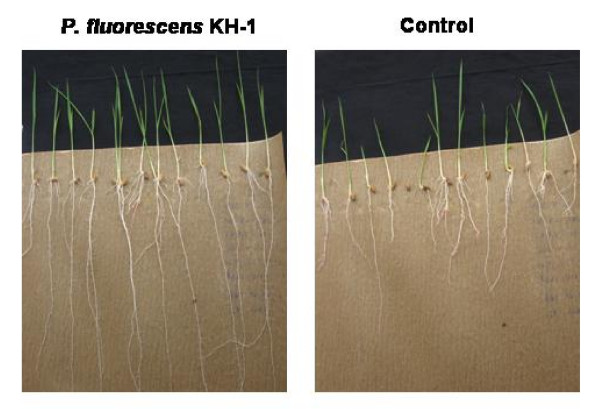
**Effect of *P. fluorescens *strain KH-1on rice growth parameters under *in-vitro *conditions**. Rice seeds primed with PGPR and observations on root length and shoot length were recorded on 7^th ^day.

Rice seeds treated with different bacterial suspensions showed improvement in plant growth parameters over untreated seeds. Among six strains of fluorescent pseudomonads, *P. fluorescens *strain KH-1 significantly increased the vigor index of rice seedlings. The increase in mean root length (25.30 cm) and shoot length (11.88 cm) was significantly higher in seedlings treated with *P. fluorescens *KH-1 compared to untreated control (Fig 1). The maximum vigor index of 3718 was observed in rice seedlings treated with KH-1 suspension and less vigor index of 1654 was recorded from untreated control. In addition, greater wet (1025.2 mg) and dry (806.4 mg) weight was recorded in *P. fluorescens *KH-1 treated seedlings where as in untreated control only 490.6 and 249.4 mg of dry and wet weight was recorded (Table 1).

**Table 1:** Effect of different Plant Growth promoting rhizobacterial strains of *P. fluorescens *on seedling growth parameters under *in-vitro *conditions.

Name of the Isolate	Root Length Mean (cm)	Shoot length Mean (cm)	Germination %	Vigour Index	Wet weight (mg)	Dry weight (mg)
KH-1	25.30^a^	11.88^b^	100	3718^a^	1025.2^b^	806.4^a^
AH-1	25.08^c^	11.32^e^	100	3640^c^	995.5^d^	793.6^b^
Pf-1	25.04^c^	11.64^c^	100	3668^b^	1057.1^a^	739.3^d^
Py-15	24.14^e^	11.40^d^	99	3518^de^	977.9^f^	738.1^e^
TDK-1	25.14^b^	11.92^a^	100	3706^ab^	1007.6^c^	806.4^a^
MDU-2	24.68^d^	11.26^f^	99	3558^d^	986.7^e^	752.7^c^
Control	17.02^f^	5.96^g^	72	1654^f^	490.6^f^	249.4^f^

Values are mean of two replications. Means in a column followed by same superscript letters are not significantly different according to Duncan's multiple range test at *P = 0.05*.

### 2-D PAGE analysis

**Figure 2:**
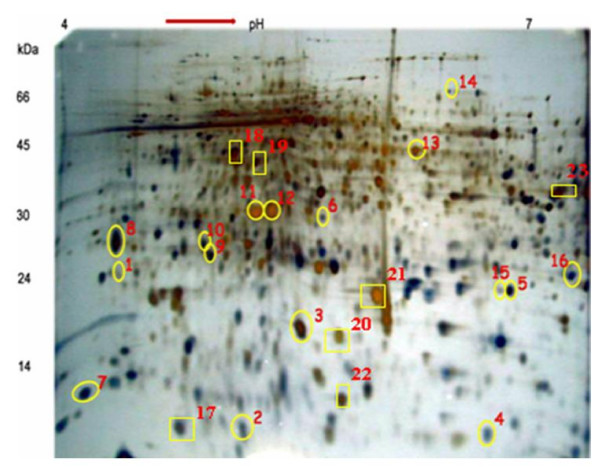
**2-D gel analyses of proteins extracted from the leaf sheath tissues of rice plants primed with PGPR**. In the first dimension (IEF), 100 μg of protein was loaded on an 18 cm IPG strip with a linear gradient of pH 4-7 and 12% SDS-PAGE gels were used in the second dimension. Proteins were visualized by silver staining. The circled spots represent the proteins that showed significant up-regulation upon PGPR priming and squares represent the significantly down-regulated proteins during PGPR priming.

Based on the previous literature in rice proteomics, we chosen 2-DE gel with pH 4-7 range and a 12% linear poly-acrylamide gel for our experiments. A total of twelve 2-DE gels were run to study the Rice-PGPR interactions, which includes three sub-replications, two treatments (PGPR treated and untreated) and two biological replications. Protein spots were reproducibly resolved in all 12 gels which results in similar protein spot locations across all the replications (Fig 2A and 2B). 2D-PAGE analysis of PGPR primed and non-primed rice leaf sheath protein revealed the differential expression of 23 protein spots which showed significant difference in their abundance between control and treated samples. Among the 23 proteins, sixteen were up-regulated and seven were down-regulated (Table 2). Most distinct six differential spots were sequenced and functionally characterized.

**Table 2:** Differential proteome analysis of PGPR primed rice sheath tissues

Spot ID	Molecular weight	Isoelectric focusing point (pI)	Change in rice protein profile due to PGPR priming
**1**	**26**	**4.33**	Up regulated
**2**	**08**	**5.16**	Up regulated
**3**	**19**	**6.22**	Up regulated
**4**	**07**	**6.30**	Up regulated
**5**	**21**	**6.44**	Up regulated
**6**	**31**	**5.35**	Up regulated
**7**	**10**	**4.26**	Up regulated
**8**	**27**	**4.24**	Up regulated
**9**	**26**	**4.82**	Up regulated
**10**	**28**	**4.75**	Up regulated
**11**	**32**	**5.21**	Up regulated
**12**	**32**	**5.45**	Up regulated
**13**	**43**	**5.00**	Up regulated
**14**	**66**	**5.45**	Up regulated
**15**	**20**	**6.35**	Up regulated
**16**	**27**	**6.81**	Up regulated
**17**	**07**	**4.61**	Down regulated
**18**	**44**	**4.89**	Down regulated
**19**	**42**	**5.23**	Down regulated
**20**	**19**	**5.74**	Down regulated
**21**	**24**	**5.92**	Down regulated
**22**	**10**	**5.67**	Down regulated
**23**	**38**	**6.90**	Down regulated

### Analysis of differentially expressed proteins

Analysis of PMF data of six proteins derived by MS analysis using MASCOT search algorithm showed homology to the following proteins 1) Putative p23 co-chaperone, 2) Probable thioredoxin h-rice, 3) Ribulose-bisphosphate carboxylase (RuBisCo) large chain precursor - rice chloroplast, 4) Nucleotide Diphosphate kinase, 5) Proteasome sub unit protein and 6) Putative glutathione S-transferase. The protein sequences were submitted to SWISSPROT and the accession No. were obtained from the Genbank organisation. The analysis showed that the protein spot 1 (Genbank accession no. P0C8Z0) has the role in energy metabolism where as protein 2 (Genbank accession no. A2YIW7) has functional role in defense and metabolism. Similarly, Protein spot 3 (Genbank accession no. P0C511), 4 (Genbank accession no. A6N0M9), 5 (Genbank accession no. P0C8Y9) and 6 (Genbank accession no. A1XBB7) have role in general metabolism, primary metabolism and defense, ISR, biotic and abiotic stress respectively (Table 3).

**Table 3:** List of expressed differential proteins in rice leaf sheath tissues in response to PGPR priming, identified through 2-DE-LC-MS/MS

Spot No	Accession number	Putative Function	Mass	pI value	Score	Number of peptides	Molecular function	Functional category
1	P0C8Z0	Uncharacterized protein OsI_027940 having higher similarity with Putative p23 co-chaperone	22836	4.33	44	3	Assembly of RuBisCO holoenzyme	Energy metabolism
2	A2YIW7	Probable thioredoxin h - rice (Phloem sap 13 kDa protein1)	13319	5.16	140	10	Methionine sulfoxide and H_2_O_2 _tolerance (Byproducts of oxygen metabolism)	Defense and metabolism
3	P0C511	Ribulose-bisphosphate carboxylase large chain precursor	53418	6.22	95	8	Photosynthesis and accumulation of chlorophyll	Metabolism
4	A6N0M9	Nucleotide DiPhosphate kinase	16835	6.30	118	6	Catalyze the exchange of phosphate groups between different nucleoside diphosphates	Primary metabolism and defense
5	P0C8Y9	Proteosome sub unit alpha type-4-2 protein	27180	6.44	84	10	degradation of proteins modified by oxidation	Induced systemic resistance
6	A1XBB7	Protein IN2-1 homolog B (Putative glutathione S-transferase GSTZ5)	27458	5.35	71	4	Recognition and transport of a broad spectrum of reactive electrophilic compounds	Biotic and abiotic stress

## Discussion

Many rhizobacteria have been reported to stimulate plant growth under different conditions [11,12,8]. Seed treatment with fluorescent pseudomonads exhibited plant growth promotion in tomato and hotpepper [13]. In the present study, six PGPR strains were tested for their efficacy to promote plant growth under *in vitro *conditions. Among the various strains evaluated, *P. fluorescens *strains KH-1 was found to be very effective in promoting seedlings growth in rice compared to other strains under *in vitro *conditions. In addition, the strain KH-1 was effective in increasing yield and reducing pest and disease incidence on rice under different ecosystems [10]. 2D-PAGE analysis of leaf sheath proteome from control and treated plants showed the over expression of RuBisCO in *P. fluorescens *KH-1 treated samples. It was reported that RuBisco plays a significant role in photosynthesis and accumulation of chlorophyll [14]. Thus, it is assumed that over expression of RuBisCO may lead to increase in the photosynthetic activity of treated plants in order to attain greater growth and possible link with plant defense.

Another PGPR responsive protein was found to be a chaperone which is known to be a stress-related protein that binds particularly to denatured proteins to prevent degradation and to assist in protein refolding of ATP [15]. In eubacteria and eukaryotic organelles, chaperonin 60 is presumably involved in numerous enzyme-folding functions [16]. In plant chloroplasts, the level of chapronin 60, being involved in assembly of RuBisCO holoenzyme, is normally coordinate with RuBiSCO [17]. However, Holland et al. [18] reported that the accumulation of chapronin 60 in *N. tabacum *seedlings against salt, cold and prolonged darkness. This protein binds hsp90 and participates in the folding of a number of cell regulatory proteins. The amino-terminal domain (N-domain) of Hsp90 represents the ATP binding site and is important for interaction with its cochaperone, p23 [19]. The differential expression in our study indicates the involvement of co-chaperones in the assembly of RuBisCO which is an important enzyme in chloroplast metabolism and photosynthesis.

The priming of rice with *P. fluorescens *KH-1 induced the over expression of Nucleoside diphosphate kinases (NDKs) which catalyze the exchange of phosphate groups between different nucleoside diphosphates [20]. NDK activities maintain equilibrium between the concentrations of different nucleoside triphosphates. The expression of plant NDPKs in response to wounding [21], heat shock [22], phytochrome B [23], UV-B light [24], oxidative stress [25] and hormones [26] has been reported by several research groups. These studies suggest that NDKs over expression in the current study might play regulatory roles in addition to their primary metabolic function.

Rice plants treated with strain KH-1 showed differential expression of proteosome sub unit alpha type-4-2 protein. The main function of the proteasome is to degrade unwanted or damaged proteins by proteolysis, a chemical reaction that breaks peptide bonds. These are known to be involved in the degradation of proteins modified by oxidation [27]. In mammalian cells, the proteasome sub-unit proteins have been shown to recognize and selectively degrade oxidatively damaged proteins, such as hydrogen peroxide-modified hemoglobin [28]. Amino acids of proteins can be modified by oxygen radicals or other activated oxygen that are produced as by-products of cellular metabolism or against abiotic and biotic stresses. Subsequently, oxidatively modified proteins can undergo chemical fragmentation or form aggregates due to covalent cross-linking reactions and increased surface hydrophobicity [29]. Thus, the expression of proteosome sub-unit proteins in the current study might involve in the cell metabolism, regulation of gene expression, and responses to oxidative stress as previously reported by Peters *et al*. [30].

Similarly, the expression of GST is known to be involved in tagging toxic endogenous substrates with GSH conjugation to transport toxic substrates into the vacuole through a glutathione pump [31]. GST has numerous roles in cellular processes with a common function, namely the recognition and transport of a broad spectrum of reactive electrophilic compounds from both exogenous and endogenous origins [32]. Many plant GST genes were reported to be auxin inducible where GTS binds auxin at the noncatalytic site or catalytic site, depending on different auxins, suggesting that GTS plays different roles in auxin function. GST has an important role in plant defense from oxidative damages caused by various biotic or abiotc stresses such as heavy metal, wounding, ethylene, ozone, and pathogen attack [32]. From the known roles of GST, it is postulated that over expression of GSTs in this study might have an essential role in the ISR by priming rice plants and protecting cells from oxidative damage.

In addition, priming of rice plants with *P. fluorescens *KH-1 induced the differential expression of thioredoxin proteins. The expression of *Arabidopsis *thioredoxin AtTRX3 in *Saccharomyces *strain EMY63 improved the methionine sulfoxide and H_2_O_2 _tolerance [33].

## Conclusion

According to the previous reports, the presumed functions of the identified proteins are related to antifungal activity, energy metabolism, photosynthesis, protein degradation and antioxidation. This strongly implies the role of *P. fluorescens *KH-1 in modulating various metabolic pathways including energy metabolism and plant defense. Further studies using detailed transcriptomics and proteomic analysis of rice-*Pseudomonas *interactions will allow us to manipulate the PGPR based crop health and yield response in rice through genetic engineering.

## Materials and methods

### Effect of *P. fluorescens *on rice plant growth

The fluorescent pseudomonad strains KH-1, Pf-1, TDK-1, MDU-2, PY-15 and AH-1 were grown separately in 100 ml of King'B broth for 48 h on a rotary shaker (150 rev min^-1^) at 28 ± 2°C. The bacterial cells were harvested and centrifuged at 6,000 rpm for 15 min and resuspended in phosphate buffer (0.01 M, pH 7.0). Cell density was adjusted using a spectrophotometer to approximately 3 × 10^8 ^cfu ml^-1 ^and used as bacterial inoculum. The bacterial suspension of *Pseudomonas *strains were prepared and tested for their plant growth-promoting activity in rice (var. Co43) using the standard roll towel method. Rice seeds soaked in 10 ml of the bacterial suspension for 2 h were blot dried, placed in wet blotters and incubated in a growth chamber for 10 days. Seeds soaked in sterile water were used as controls. The vigour index was calculated using the following formula: vigour index = percent germination × seedling length (shoot length + root length) [34]. The experiments were repeated twice and each treatment had five replications.

### Preparation of talc based formulation of *P. fluorescens *strain KH-1

A loopful of *P. fluorescens *KH-1 was inoculated into the King's B broth and incubated in a rotary shaker at 150 rpm for 72 h at room temperature (28 ± 2°C). After 72 h of incubation, the broth containing 9 × 10^8 ^cfu ml^-1 ^was used for the preparation of talc-based formulation. To the 400 ml of bacterial suspension, 1 kg of the purified talc powder (sterilized at 105°C for 12 h), calcium carbonate, 15 g (to adjust the pH to neutral) and 10 g carboxy methyl cellulose (CMC) (adhesive) were mixed under sterile conditions following the method described by Nandakumar *et al*. [35]. The product was shade dried to reduce the moisture content below 20 per cent and then packed in a polypropylene bag and sealed. At the time of application, the population of bacterium in talc formulation was checked to 2.5 to 3 × 10^8 ^cfu/g.

### Treating rice plants with *P. fluorescens*

Paddy seeds (cv Co-43) were surface sterilized by 50% bleach and rinsed with water. The surface sterilized seeds were soaked in double the volume of sterile distilled water containing talc-based formulation (10 g kg^-1 ^of seed). After 24 h, the suspension was drained off and the seeds were dried under shade for 30 min [35]. Treated seeds were kept for germination in Petri dishes at 28°C in the dark. Germinated seeds were placed in 16 cm diameter pots with required spacing. Thirty days after sowing, the seedlings were carefully transplanted to individual pots about 6 hills/pot (2 seedlings/hill). Before transplanting, seedlings were treated with PGPR by seedling dip method [the roots were dipped in water containing talc formulation (20 g l^-1^) for 2 h] without any mechanical damage. Twenty days after transplantation 5 gms of Talc based bioformulations was applied to each pot as soil application.

### Sampling

Sampling of leaf sheaths was done from both control and treated plants at 48 h after soil application of *P. fluorescens *strain KH-1 and used for proteomic analysis. The experiment was repeated twice (biological replications) with adequate replications. From each biological replication, sampling of leaf sheaths was done (three replications) and protein extraction was done separately. After extraction, proteins isolated from respective treatments were combined and pooled protein sample was distributed equally into three aliquots which served as sub-replications of the pooled protein sample.

### 2D PAGE analysis

#### Protein extraction

Frozen leaf sheaths were ground in a mortar using liquid nitrogen and suspended in 10% trichloracetic acid (TCA) in acetone with 0.07% dithiothreitol (DTT) and kept at 20°C for 1 h, followed by centrifugation for 15 min at 35,000 g. The pellets were washed once with ice cold acetone containing 0.07% DTT at -20°C for 1 h and centrifuged again for 15 min at 35,000 g. This washing step was repeated four to five times until the supernatant was clear (free of chlorophyll). The final precipitated pellet was lyophilized for 2 hrs. About 10 mg of the dried powder was used for protein extraction by dissolving in 350 μL of sample lysis buffer containing 7 M urea, 2 M thiourea, 4% 3- [(3-cholamidopropyl) dimethylammonio]-1-propanesulfonate (CHAPS), 0.5% ampholytes (Bio-Rad) and 0.7% DTT. Protein extraction was done at 37°C with occasional vortexing. After 1 hr incubation, cell debris were pelleted by centrifuging for 30 min at 35,000 g at room temperature. The supernatant was distributed in 100 μL aliquots and kept at -80°C before 2D-PAGE analysis [36]. The protein content was determined by Bradford method.

#### 2D-PAGE

Equal amount of protein (100 μg) from control and treated samples were separated by 2D-PAGE. In the first dimension, IPG strips of 17 cm length and pH 4-7 were used. Electrophoresis was carried out at 500 V for 1 h, followed by 1000 V for 1 h and 2950 for 24 h. After IEF, the proteins were separated by SDS-PAGE in the second dimension using 12% polyacrylamide gels [37]. The gels were stained by silver staining method. For each biological replicate, one set of gels with high resolution running at different times were selected for further analysis. Relative abundance of protein spots was quantified with Melanie III (GeneBio, Geneva, Switzerland) after silver staining the gels and scanning with a densitometry (GS-700, Bio-Rad).

### Protein identification

#### Protein digestion

Differential protein spots were excised from preparative gels. The excised protein spots were digested with trypsin using the MassPREP station (Waters). The excised spots were de-stained with 50 μL of 50 mM ammonium bicarbonate and 50 μL of 50% acetonitrile, washed once with 50 μL of 100 mM ammonium bicarbonate and 50 μL of dehydrated acetonitrile. Digestion was done with 6 ng μL^-1 ^trypsin in 25 μL of 50 mM ammonium bicarbonate for 5 h at 37°C. The digested protein was extracted twice [first with 1% formic acid (30 μL), and second with 1% formic acid (12 μL)/50% acetonitrile (12 μL)]. The digested proteins were combined and maintained in a PCR plate at 4°C for further analysis.

#### Protein identification and sequencing by 2D Nano LC MS/MS

Protein identification and sequencing was carried out using two dimensional liquid chromatography ESI MS (Agilent 1100 series 2DnanoLC MS). Tryptic digested protein was subjected to column followed by reverse phase separation. Peptides get ionized in the liquid phase in the Electrospray ionizer and enter the ion trap, get fragmented (MS/MS) and detected. The data has been sent to MASCOT search engine (Agilent) for analysis.

#### Database Searching with MS/MS Spectra

MS/MS spectra were used to search against the NCBI non- redundant protein database using MS/MS Ion Search Engine, a computer software program conducting protein identification based on matching the MS/MS spectra of a protein with a protein or DNA sequence data base http://www.matrixscience.com/search_form_select.html. The significance of the protein match with the ion score was based on the Mouse scoring algorithm [38]. The ion score was calculated as -10 × LOG10(P), where P is the absolute probability that the observed match is a random event. Thus, a relatively small P value means that the match of identified protein and the MS/MS spectra is not a random event. A significant specific match increases the ion core, so a high score means highly significant matching (MASCOT Help; http://www.matrixscience.com/help/scoring_help.html). A single protein having a higher score than the minimum score for the significance level (p < 0.05) was judged as a significant match. In each MASCOT search output result, the minimum score for significance level was provided, based on the absolute probability and the size of the sequence database being searched.

## Competing interests

More than five hundred PGPR strains have been isolated from various regions of India and their growth promotion and biocontrol ability has been tested in several crops by various research scholars of Lab ≠ 20, Department of Plant Pathology, Tamil Nadu Agricultural University, Coimbatore, India. Results have been published in various peer reviewed journal. This the first study on analyzing the molecular response of PGPR primed crop plant. This will be the base to progress further research at molecular level.

## Authors' contributions

KS, MR and RS have made significant contributions to design the objective and experiments. KS and LK were carried out the protein separation. KS and MR were potentially contributed the protein identification and interpretation of data. RS, TR and SS mobilized the necessary lab chemicals. PB provided facility. KS, DS, MR and RS have involved in drafting the manuscript or revising it critically for important content. All authors were significantly contributed and approved the final manuscript.
